# In Memoriam Dra. Charlotte Dravet (1936–2025)

**DOI:** 10.31083/RN44103

**Published:** 2025-08-27

**Authors:** Rosa Peraita-Adrados, Javier Salas-Puig

**Affiliations:** ^1^Unidad de Trastornos del Sueño de Neurofisiología Clínica, Hospital General Universitario e Instituto de Investigación Biomédica Gregorio Marañón, Universidad Complutense de Madrid (UCM), 28040 Madrid, España; ^2^CDINC-Instituto Universitario de Neurología, 08021 Barcelona, Spain; ^3^Policlínica San Francisco, 33007 Oviedo, Asturias, Spain

**Figure:**
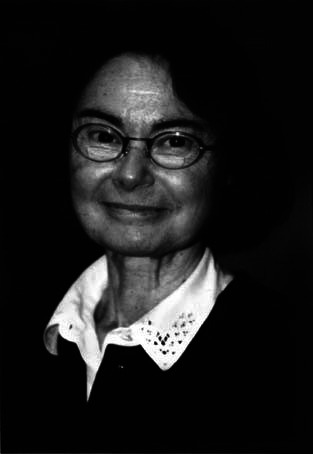
Dra. Charlotte Dravet (1936–2025)

El pasado 9 de mayo ha fallecido a la edad de 88 años la Dra. Charlotte Dravet en su Marsella natal. Licenciada en Medicina por la Universidad de Aix-Marseille, y especialista en Pediatría, fue en el Centro Saint Paul de Marsella (Centro de la OMS para “La infancia y la adolescencia inadaptada”, actualmente Hospital Henri Gastaut) donde la Dra. Dravet, bajo la dirección del Prof. H. Gastaut y el Dr. J. Roger [1], realizó a partir de 1965 su tesis Doctoral titulada: “Encéphalopathie Épileptique de l’Enfant avec Pointe-Onde lente diffuse (“petit mal variant”)” en la que estudió las características electroclínicas de 50 pacientes. Un año más tarde H. Gastaut y Ch. Dravet junto con otros autores del Hospital de la Timône y del Centro Saint-Paul, publicaron la serie de 100 pacientes con las mismas características electroclínicas proponiendo el epónimo de síndrome de Lennox [2]. Tres años más tarde se propuso el término de síndrome de Lennox-Gastaut vigente en la actualidad. En 1971 obtuvo la especialidad de Psiquiatría. Fue directora asociada del Centro Saint-Paul desde 1989, hasta su jubilación en el año 2000, y vivió en el propio Centro con una dedicación total a los enfermos y familiares.

En 1978 Charlotte Dravet describió la epilepsia mioclónica grave del lactante y, en 1981, la epilepsia mioclónica benigna del lactante al analizar con minuciosidad las características clínicas y electroencefalográficas de series de pacientes que tenían fundamentalmente crisis mioclónicas, unos con muy mal pronóstico y otros, al contrario, con buen pronóstico de las crisis epilépticas. Otros grupos confirmaron la utilidad en la distinción de ambos síndromes que finalmente fueron aceptados y descritos con precisión ya en la primera edición de la llamada “Guía Azul” de las epilepsias en 1984 [3]. Diferentes series de pacientes con inicio de las crisis en los dos primeros años de vida no solamente mioclónicas, con especial sensibilidad a la temperatura (fiebre) y frecuentemente con fotosensibilidad—descritos en Verona por Bernardo Dalla Bernardina y en el propio Centro Saint-Paul—provocaron que en pocos años la epilepsia mioclónica grave del lactante pasó a denominarse síndrome de Dravet. En 2001 se descubrió la genética de la enfermedad debida a una mutación en el gen SCN1A y de forma definitiva se dio a conocer en todo el mundo. Disponer de un diagnóstico genético y de nuevos fármacos siguen generando un gran interés tanto a nivel de investigación básica como a nivel clínico con el impulso que supone disponer de nuevos tratamientos.

Co-editora de libros importantes sobre Epilepsia y autora de numerosos artículos de los que destacamos tres, “Severe Myoclonic Epilepsy in Infants and its Related Syndromes” [4], “The Core Dravet Syndrome Phenotype” [5] y el libro sobre el síndrome de Dravet co-editado con Renzo Guerrini [6].

Sus contribuciones científicas en Epilepsia han sido reconocidas internacionalmente y en 2011 Francia la condecoró como Caballero de la Legión de Honor. Posteriormente la Liga Internacional contra la Epilepsia en el congreso de Barcelona en el año 2017 la concedió el Premio a la Trayectoria Vital.

Fue presidenta de la Liga Francesa contra la Epilepsia (1997–1999), miembro de la Task Force on Classification and Terminology de la Liga Internacional contra la Epilepsia (1996–2004), y organizadora del primer Día Internacional de la Epilepsia en Francia en el año 2000.

Después de su jubilación continuó con su actividad clínica y docente como Consultora honoraria en el Policlínico A. Gemelli, de la Universidad Católica del Sagrado Corazón en Roma, y participando en reuniones de asociaciones de pacientes en todo el mundo. En Madrid, participó en la Fundación Síndrome de Dravet, en septiembre de 2019, dedicando mucho tiempo a los pacientes y familiares.

Trabajadora incansable, perfeccionista, dedicada por entero a los pacientes, generosa y empática, muy asequible para los numerosos especialistas del mundo entero que tuvimos la suerte de formarnos con ella y el equipo del Saint-Paul. Nunca olvidaremos la capacidad de transmitir su pasión por el conocimiento de las epilepsias sobre todo de la edad infantil.

Descanse en paz.

Rosa Peraita-Adrados, Javier Salas-Puig

## Contribuciones de los Autores

RPA y JSP contribuyeron a la redacción de la necrológica y a los cambios editoriales del manuscrito. Ambos autores leyeron y aprobaron la versión final del manuscrito. Ambos autores han participado de manera suficiente en el trabajo y han acordado asumir la responsabilidad de todos los aspectos del mismo.
